# Mentoring the next generation of physician-scientists in Japan: a cross-sectional survey of mentees in six academic medical centers

**DOI:** 10.1186/s12909-015-0333-2

**Published:** 2015-03-19

**Authors:** Ken Sakushima, Hiroki Mishina, Shunichi Fukuhara, Kenei Sada, Junji Koizumi, Takashi Sugioka, Naoto Kobayashi, Masaharu Nishimura, Junichiro Mori, Hirofumi Makino, Mitchell D Feldman

**Affiliations:** 1 Hokkaido University Graduate School of Medicine, Sapporo, Japan; 2 Department of Healthcare Epidemiology, Kyoto University School of Public Health, Sapporo, Japan; 3 Department of Healthcare Epidemiology, Kyoto University School of Public and Health Center for Innovative Research for Community and Clinical Excellence, Kyoto, Japan; 4 Department of Medicine and Clinical Science, Okayama University Graduate School of Medicine, Dentistry and Pharmaceutical Science, Okayama, Japan; 5 Department of General Internal Medicine, Kanazawa University Hospital, Kanazawa, Japan; 6 Saga University Community Medical Support Institute, Saga, Japan; 7 Ehime University Medical Education Center, Toon, Japan; 8 Department of Respiratory Medicine, Hokkaido University Graduate School of Medicine, Sapporo, Japan; 9 Department of Medical Education, Shinshu University, Matsumoto, Japan; 10 Department of Medicine and Associate Vice Provost for Faculty Mentoring at the University of California, 1545 Divisadero, Suite 315, San Francisco, CA 94143-0320 USA

**Keywords:** Mentoring/mentorship, Medical education-career choice, Medical education-postgraduate, Physician-scientist

## Abstract

**Background:**

Physician-scientists play key roles in biomedical research across the globe, yet prior studies have found that it is increasingly difficult to recruit and retain physician-scientists in research careers. Access to quality research mentorship may help to ameliorate this problem in the U.S., but there is virtually no information on mentoring in academic medicine in Japan. We conducted a survey to determine the availability and quality of mentoring relationships for trainee physician-scientists in Japan.

**Methods:**

We surveyed 1700 physician-scientists in post-graduate research training programs in 6 academic medical centers in Japan about mentorship characteristics, mentee perceptions of the mentoring relationship, and attitudes about career development.

**Results:**

A total of 683 potential physician-scientist mentees completed the survey. Most reported that they had a departmental mentor (91%) with whom they met at least once a month; 48% reported that they were very satisfied with the mentoring available to them. Mentoring pairs were usually initiated by the mentor (85% of the time); respondents identified translational research skills (55%) and grant writing (50%) as unmet needs. Mentoring concerning long-term career planning was significantly associated with the intention to pursue research careers, however this was also identified by some mentees as an unmet need (35% desired assistance; 15% reported receiving it).

**Conclusions:**

More emphasis and formal training in career mentorship may help to support Japanese physician-scientist mentees to develop a sense of self-efficacy to pursue and stay in research careers.

## Background

Physician-scientists, defined as persons with an MD or equivalent degree whose major professional activity is to conduct clinical research [1], play an important role as clinician-investigators in academic biomedical research. In spite of this importance, some commentators have warned that the pipeline of clinical researchers in the U.S. face a number of significant challenges that threaten them with becoming an “endangered species [2,3]”. These challenges include obtaining adequate grant support, protected time for research, and obtaining adequate mentoring among others [2,4-8]. As a result, over the past decade many commentators have expressed concern about a “leaky academic research career pipeline”, further exacerbated by the aging of the independent grant-awarded workforce in the U.S. [4] and globally [9,10]. Further, less exposure to academic medicine and research in the early phases of physician training has led to fewer medical school graduates choosing academic careers [11].

Similar challenges have emerged in Japan over the past decade [10]. As in the U.S., fewer MDs are choosing academic research careers [12] and Japanese clinical research productivity has declined compared with basic and life science research productivity [13]. The medical research career pipeline in Japan is populated by trainees who have completed a 6-Year MD degree and then undertake a 4-year PhD course in a graduate school of medicine [14]. These graduate students (equivalent to research fellows in the US system) are assigned to either clinical departments, or basic/social science departments. In clinical departments, all faculty, post-doctoral fellows, and graduate students are required to provide clinical care, which graduate students manage concurrently with their research activities [15-17].

Although many young Japanese physicians-in-training articulate a desire to pursue academic research careers early in training [18], their interest often wanes during residency or post-graduate training [10,12,19]. A recent analysis of career trends of physician-scientists in Japan found that while the overall numbers of physician-scientists has remained stable, the number of younger physician-scientists declined sharply from 1996 to 2008 [10]. Why this shift occurred is not clear. In the US, much attention has focused on mentorship as a key ingredient to support recruitment and retention of trainees and junior faculty in biomedical research careers [7,11,20-23]. Mentorship also plays an important role in supporting trainee and junior faculty career development, satisfaction, and productivity [24,25]. However, the extent to which mentorship (or the lack of robust mentoring relationships) may be contributing to the diminishing interest in academic research careers among young physicians in Japan is not known. In fact, there has been very little prior research on mentorship in academic medicine in Japan, and no prior study has attempted to examine and describe mentoring relationships between physician-scientist trainees and faculty in Japanese academic health sciences institutions. In this project, we surveyed physician-scientist trainees at 6 academic medical centers in Japan to understand their current mentoring relationships and perceived mentoring needs.

## Methods

### Design, setting and participants

In November 2011, we mailed by post an anonymous Japanese language questionnaire survey to 1700 potential mentees (physician-scientists as well as non-MD biomedical graduate students) at 6 academic medical centers in Japan. We selected these medical centers on the basis of geographic diversity and to represent a cross-section of academic medical centers in Japan. All post-graduate trainees in the schools of medicine at each of the 6 universities were considered eligible to receive the survey. We limited the current analysis to respondents with an MD degree in order to focus on mentorship of physician-scientists. The questionnaires were collected by our co-investigators at each of the 6 centers and were sent to the Nippon Research Center (http://www.nrc.co.jp) in Tokyo, Japan for input. The data was analyzed at Kyoto University. The study was reviewed and approved by the Kyoto University Graduate School and Faculty of Medicine, Institutional Review Board and Ethics Committee on October 5th, 2011, reference number E1241.

### Terminology

While there is a long tradition of senior-junior professional relationships in Japan [26,27] there is no exact translation for the word “mentor”, “mentee”, or “mentoring” in Japanese. These are borrowed or imported terms that may not be understood the same way in Japan as they are in the US or in Europe [26]. For purposes of this research we created the following operational definition of mentoring, which appeared at the beginning of the questionnaire: “A mentor is defined as a faculty member who uses his/her knowledge, skills, and experience to give individual guidance to a mentee to help the mentee pursue their own research”.

### Questionnaire and sampling

The Japanese language questionnaire items were based, in part, on a study of mentorship in academic medicine in the US that focused on mentoring of junior faculty [24]. The Japanese survey consisted of 35 items covering: demographic characteristics, department and area of research (basic medicine, clinical medicine, social medicine or other); mentorship characteristics, including mentor assignment, position of mentor, department of mentor and mentoring frequency; the mentee’s perceptions of mentoring, including satisfaction with mentoring. Questions about mentoring and mentor-mentee relationships were restricted to the person who, in the opinion of the mentee, spent the most time and effort with him or her. Questions about career and intention to pursue research included such items as expected graduation date, intention to stay at their current institution, intention to continue research activities, financial sufficiency, and current percentage of time devoted to research activities. Survey questions were mainly multiple choice with some items formatted for free-text responses. At each university we assigned a faculty member to coordinate the distribution and collection of the questionnaires. Two postal reminders were sent to participants to complete the questionnaire. No incentives were offered for completion. Completion of the anonymous questionnaire constituted consent to participate in the study.

### Data analysis

Descriptive statistics were used to characterize respondent physician-scientists and their mentorship experiences. Comparison of mentoring topics provided to mentees versus topics desired by the mentees but not provided as well as intention to pursue a research career were examined using bivariate and multivariate analyses. Evaluations of categorical variables of this association were assessed with chi-square tests, Fisher Exact Test and risk ratios with a significance level set at p < 0.05. A generalized linear model with log-binomial regression was used to estimate adjusted risk ratio [28]. All statistical analyses were conducted with STATA version 12.0 (STATA Corporation, College Station, TX, USA).

## Results

### Participant demographics

A total of 683 of 1700 (40%) potential mentees from the 6 medical centers responded to the survey. Among respondents, 428 (63%) reported that they had an MD degree and were classified as physician-scientists for purposes of this analysis. Of these, 24% were female; mean age was 33 years. Demographics of physician-scientists with and without a mentor are described in Table 1. The majority of male (90.1%) and female (93.8%) physician-scientists reported that they had a mentor. Of the variables we examined, including gender, age, area of research, and intention to pursue a research career, only time allocation for research activities was significantly associated with having a mentor.

**Table 1:** **Demographics of physician-scientist respondents in Japan with a mentor (N=389) and without a mentor (N=39)**

Demographics	Have mentor, n (%)		p*	Fisher exact test p
Gender				
Male	283	(90.1)	0.25	0.33
Female	105	(93.8)		
Age (y)				
<40	371	(91.4)	0.65	0.65
40 or more	15	(88.2)		
Department				
Clinical medicine	325	(90.3)	0.28	0.40
Basic science	46	(93.9)		
Other	18	(100)		
Area of research theme				
Clinical research	165	(94.3)	0.54	0.79
Basic science	200	(95.2)		
Other	19	(100)		
Time allocation for research activities				
0-20%	72	(74.2)	<0.01	<0.01
21-40%	129	(94.2)		
41-60%	77	(97.5)		
61-80%	83	(96.5)		
81-100%	26	(100)		
Intention to pursue research career				
Yes	115	(95.0)	0.07	0.09
No/Uncertain	272	(89.5)		

Note: Due to missing data, responses do not always total to 389.

### Mentorship demographics

Mentorship demographics of physician-scientist mentees are shown in Table 2. Most mentees (91%) had a mentor in their same department and the mentor was mainly at the full Professor/Chair of the department or Associate Professor rank. Most mentees (average of male and female = 77%) reported that they met with their mentor at least once a month. More men (76%) then women (73%) reported that overall they were satisfied with the mentoring available to them, but this difference was not statistically significant.

**Table 2:** **Mentorship characteristics of survey respondents with a mentor**

Mentorship demographics	No. (%)			
	(N=389 with mentor)			
	Male	Female	p	Fisher Exact Test
Department of mentor				
Same department	255 (91.1)	95 (91.3)	0.93	1.0
Another department	25 (8.9)	9 (8.7)		
Position of mentor				
Dept. Chair / Professor	67 (23.9)	22 (21.4)	0.62	0.66
Associate Prof.	59 (21.1)	27 (26.2)		
Lecturer	58 (20.7)	19 (18.4)		
Assistant Prof.	80 (28.6)	32 (31.1)		
Other	16 (5.7)	3 (2.9)		
Mentor assignment initiated by				
Mentor	242 (87.7)	84 (88.4)	<0.01	0.01
Mentee	34 (12.3)	11 (11.6)		
Other	4 (1.4)	8 (7.8)		
Mentoring Meeting Frequency				
Every month or more	213 (76.1)	83 (79.8)	0.44	0.50
Other (combining all other categories)	67 (23.9)	21 (20.2)		
Satisfaction with Mentoring				
Very satisfied/ A little satisfied	212 (75.7)	75 (72.8)	0.56	0.60
Not Satisfied\Neutral	68 (24.3)	28 (27.2)		

### Mentoring content

Respondents noted both congruencies and disparities in terms of the substantive content they desired from their mentoring relationships, and what they reported they actually received from their mentors (Table 3). Most respondents reported that they received adequate mentoring in research design (85%), research management (89%), and assistance with presentation and posters (75%). Topics with the widest apparent disparities between what mentees reported that they received and desired from their mentor included manuscript preparation/publishing (90% desired assistance but only 60% reported having received it), translational research skills (55% desired assistance but < 10% reported having received it) grant writing (50% desired assistance but < 20% received it) and long-term career planning (35% desired assistance but 15% received it). Interestingly, discussion of balancing personal and professional demands was neither very much desired by mentees nor delivered by most mentors.

**Table 3:** Topics reported by respondents as a received or as a desired mentoring need*

Topic	Received by mentee N (%)	Desired by mentee N (%)
Manuscript preparation/publishing	236 (61)	352 (90)
Research Design	337 (87)	350 (90)
Research management	346 (89)	340 (87)
Presentation/posters	268 (69)	296 (76)
Computer skills/statistical skills	130 (33)	282 (72)
Translational research skills	34 (9)	211 (54)
Grant writing	71 (18)	195 (50)
Networking nationally and internationally	64 (16)	189 (49)
Obtaining funding	56 (14)	173 (44)
Clinical care	144 (37)	162 (42)
Networking on campus	71 (18)	135 (35)
Long-term career planning	61 (16)	134 (34)
Teaching	20 (5)	124 (32)
Time management	62 (16)	97 (25)
Developing an educator’s portfolio	38 (10)	96 (25)
Understanding promotion and tenure	14 (4)	71 (18)
Balancing personal/professional demands	87 (22)	66 (17)
Communicating effectively with colleagues	63 (16)	61 (16)
Preparation of Curriculum Vitae	6 (2)	55 (14)
Review promotion/merit packet	12 (3)	47 (12)

*Denominator of N=389 was used for calculation of % in both columns.

## Discussion

Researchers and policymakers are increasingly concerned about an observed and persistent decrease in the number of early career physician-scientists in Japan who choose and/or ultimately remain in research careers. In an effort to investigate factors that might explain and/or help to reverse this phenomenon, this study is the first to describe mentoring relationships and the content of mentoring for junior physician-scientists in Japan. Contrary to our expectations, we found that most physician-scientist trainees reported that they had a mentor and about half reported that they were very satisfied with the mentoring available to them. Unlike some research from the US [24,25], we did not identify a significant gender gap in access to mentors or in satisfaction with mentoring. As has been reported previously from the US [24], we did find that there was a significant association between having a mentor and more time spent conducting research.

Unlike in the US where some research suggests that mentors are most likely to be self-identified by the mentee [21], our data suggests that most mentoring relationships were initiated by the mentor. This is not surprising in light of the more hierarchical nature of professional relationships in Japan [26] and the fact that almost one-quarter of the mentors were at the professor rank, which in Japan is often indicative of being the department chair.

Our findings must be viewed through a cultural lens, recognizing that although the very terms “mentor” and “mentoring” have no exact equivalent in Japanese, there is a rich tradition of junior/senior (or *senpai*/*kohai*) professional relationships in Japan. These share some similarities but also deep cultural differences from the Western concept of mentorship. The Japanese concept encompasses both a more personal and more rigidly defined relationship in which the senior *senpai* must guide the junior *kohai* in the customs and behaviors required to be successful in the organization. The *senpai’s* role is not so much to nurture independence in the *kohai* as it is to protect him or her in the organization. What the *senpai* receives in return is unquestioned loyalty and hard work [27]. These traditional expectations are likely carried over to the mentoring relationships in academic medical settings in Japan where the senior mentor may often view their role as one in which they will guide and protect the junior scientist, but *not* nurture their independent research careers. In contrast, the focus of effective research mentoring in the US is to promote the transition to independent careers for the mentee [7].

As seen in Table 3, mentees report discussing a broad variety of topics with their mentor. Given the imperative to publish it is not surprising that these mentees most frequently reported that they desired assistance from their mentor with manuscript preparation and publishing; somewhat surprisingly only about two-thirds reported having received this assistance. However, the equally important skills of research design and research management were both frequently desired by mentees and addressed by the mentors.

Several key topics are reportedly discussed infrequently with mentors although mentees indicated that they desired assistance with them. For example, although 50% of mentees reported wanting assistance from their mentor in grant writing and 44% in obtaining funding, only 18% and 14%, respectively, said that this was discussed with their mentor. Likewise, most reported that they wanted assistance in computer and statistical skills and a third wanted assistance in long-term career planning, but these topics were infrequently addressed in the mentoring meetings. It is clearly challenging for early career physician-scientists to progress in their careers as researchers with inadequate training in these key skills and without explicit discussions with their mentors about long-term career planning.

While our findings suggest that access to mentorship is not a barrier for early career physician-scientists in Japan, the content of the mentoring discussions does not appear to adequately address their needs. Future research in Japan should address these gaps in mentor knowledge and competencies. Recent research from the US has begun to shed light on the qualities of outstanding mentors [29], the core competencies of research mentors, and how to measure competencies and provide feedback [22,30-33]. The challenge for Japanese educators and policy makers is how to apply these findings and emerging instruments to the Japanese context so as to build on the strengths of the mentoring relationships in Japan while respecting cultural norms and traditions.

As reported in Table 1, we were surprised to find that the majority of the early career physician-scientist mentees in our survey reported that they did not have an intention to pursue a research career, and that there was no difference between those with and without a mentor. A vital skill for a research mentor is to understand and support their mentee’s sense of self-efficacy to pursue a research career, which in turn impacts their intention to successfully pursue this career. Perceived self-efficacy is a strong predictor of behavior; people tend to avoid activities that they believe they cannot carry out, and engage in activities they judge themselves capable of handling [34,35]. The social cognitive career theory (SCCT) [36] demonstrates that person-environment interactions from learning experiences influence perceived career self-efficacy and career-specific outcomes, including the academic path toward a scientific career goal [36].

Our findings that most mentees in our survey have an identified mentor provide some cause for optimism. The next steps would be the implementation of a national program to train research mentors in Japan according to a model such as the National Research Mentoring Network recently announced by the U.S. National Institutes of Health. Such a program can help to incorporate “high touch” career development at each stage of training in order to support early career physician-scientists’ intentions to pursue a research career. However, our results also highlight clear challenges resulting from the current research-training framework, as well as identified mismatches in the mentoring content that trainee’s desire, and that which is currently delivered.

Our findings should be interpreted in light of some limitations. First, Using a cross sectional design, participants were drawn from 6 of the 51 public academic graduate schools of medicine in Japan. This design allowed us to gather a broad snapshot of mentoring in Japan but limited our ability to understand the causal relationships between mentoring and other outcomes of interest. Second, our findings are limited by the fact that we surveyed only mentees, not their mentors. Research on mentors in Japan will help to provide a fuller picture of mentoring relationships. Third, the low response rate and our lack of data on non-respondents also may limit the generalizability of our findings. The fact that mentorship is a relatively new concept in Japan may have dissuaded some participants from completing the survey. Finally, this study focused on mentorship in the context of research careers. We did not evaluate the reasons that physician-scientists did not complete graduate school. Our results therefore represent characteristics of potential mentees who stay in graduate school and have an opportunity to pursue their research. However, because the educational framework of all graduate schools of medicine are established at the national level, and there is a high degree of consistency among institutions, our results are likely generalizable across similar institutions in Japan.

## Conclusions

In conclusion, we found that mentoring is common in academic medical research centers in Japan, but that many of the early career physician-scientists receiving mentoring did not intend to pursue research careers after graduation. More emphasis on career mentorship and formalized mentor training in Japan will provide mentors with the knowledge and skills to help their mentees to develop the sense of self-efficacy required to pursue careers in research.
